# Gyro-Mag: Attack-Resilient System Based on Sensor Estimation

**DOI:** 10.3390/s25072208

**Published:** 2025-03-31

**Authors:** Sunwoo Lee

**Affiliations:** Department of Information Security, Seoul Women’s University, 621, Hwarang-ro, Nowon-gu, Seoul 01797, Republic of Korea; sun.lee@swu.ac.kr

**Keywords:** resilience, IMU system, signal injection attack, attack detection

## Abstract

Several researchers recently demonstrated that attackers can interfere with an inertial measurement unit (IMU) sensor’s normal function or take complete control of sensor measurements by physically injecting malicious signals into the sensor. Although there are existing methods for detecting such signal injection attacks, most do not provide resilience. Indeed, detection-only methods cannot respond when attacks have already occurred, which results in accidents such as crashes or falls. In this paper, we propose the first method that can detect signal injection attacks on IMU sensors based on the relation between the gyroscope and the magnetometer, and provide long-term resilience against these attacks. We construct a mathematical model to estimate one sensor’s data from the other’s data based on their relation. With this mathematical model, the device can detect signal injection attacks on the IMU sensor and continue to function in a near-normal state based on the estimated data. Our method can be easily adapted to deployed devices since it requires only estimation software and no additional hardware. We evaluated our method using a total of five open datasets and commercial devices. Our method has a resilience of 99.78% against signal injection attacks while consuming only reasonable computational costs.

## 1. Introduction

A sensor is an electronic device designed to detect changes in the environment, such as light, sound, or movement. Through these sensors, embedded devices interact with physical quantities in the real world. In particular, IMU sensors specifically measure dynamic motion through gyroscopes that measure torsional or rotational motion and magnetometers that measure the magnetic field of the Earth by surveying the magnetic flux density in the air. Therefore, IMU sensors can provide many services when mounted in industrial and general household devices. For example, they are used to track motion in consumer electronics like smartphones, video game remote controllers, and robotic vacuums. They are even used to steer aircraft with or without a human pilot. Moreover, IMU sensors are available in small 20 μm to 1 mm sizes and offer low power consumption and costs as well as high reliability and robustness. For these reasons, the demand for IMU sensors has increased rapidly in recent years. Mordor Intelligence stated that the global market is expected to grow to USD 18.88 billion in 2026, up from USD 10.92 billion in 2020, registering a compound annual growth rate of 8.71% during the forecast period of 2021–2026 [1].

However, several researchers recently demonstrated that attackers can interfere with a sensor’s normal function or even take complete control of sensor measurements by physically injecting malicious signals into the sensor. Son et al. first performed a simple attack and injected acoustic signals into the built-in gyroscopes of drones to make movement uncontrollable [2], and Wang et al. attacked gyroscopes in a virtual reality headset and a self-driving car [3]. Further, Tu et al. showed that gyroscopes can be controlled in a much more granular way, specifically demonstrating that it is possible to mount signal injection attacks from a distance of 7.8 m that lasts up to one minute in duration [4].

Since IMU sensors are built into various devices and potentially provide a number of services, normal service provision may be impossible and property or personal damage could even occur if there were signal injection attacks. Accordingly, some researchers have studied methods to defend against these attacks, and these methods can be divided into hardware-based and software-based methods. Hardware-based methods are difficult to apply to already deployed devices because they rely on shielding [2,3,4,5] or require additional sensors for attack detection [2,3,4]. In addition, there are sensor fusion methods [3,4,6] that cover software-based methods. But it has been recently demonstrated that signal injection attacks can infiltrate these methods [7]. Alarmingly, all existing hardware- and software-based methods focus only on attack detection and do not consider resilience to signal injection attacks. Resilience is the system’s ability to sustain its intended function and trajectory despite signal injection attacks. That is, even if an attack occurs, the system should continue to operate as expected without significant deviation from its intended motion. Resilience is crucial because detection alone is insufficient. Without an effective recovery mechanism, the system remains vulnerable and may fail to function properly after an attack.

The hardware-based methods merely prevent malicious signals from being injected via shielding, or they detect malicious signals via additional sensors such as microphones. Additionally, the software-based sensor fusion method is also incapable of providing resilience because it compares and analyzes sensor data in the current state rather than estimating future sensor data. If a system can only detect signal injection attacks but cannot respond to them, it may still fail to function normally, leading to crashes or falls. Accordingly, there should be a method that can both detect and respond to these attacks (i.e., resilience). As the need for resilience increases, more researchers are working to develop methods that detect signal injection attacks and provide resilience by modeling sensor systems [8]. However, such methods have limitations—they require highly accurate sensor models and provide resilience for only about 10 s. Since recent signal injection attacks can last up to one minute [4], this duration is, unfortunately, insufficient.

In this paper, we propose the first attack-resilient IMU system based on the sensor relation that can provide long-term resilience against signal injection attacks. In other words, even if a signal injection attack occurred, the device would be able to detect the attack and behave in a near-normal state based on the data estimated through the proposed mathematical models.

Our contributions are summarized as follows:

- Our method is the first attack-resilient IMU system based on the intrinsic relation between the gyroscope and the magnetometer, addressing the limitations of prior approaches in three key ways: (1) Our approach introduces a mathematical model that estimates gyroscope data from magnetometer data. This model operates without requiring additional hardware and incurs only reasonable computational costs, making it practical for real-world deployment. (2) Unlike prior methods that only detect attacks, our approach actively recovers sensor functionality, ensuring continued operation. (3) Our method provides resilience against long-duration attacks, effectively mitigating attacks lasting up to one minute, which significantly improves robustness against prolonged threats.
- We evaluate our attack-resilient method using five open datasets as well as a commercial smartphone and IMU sensors. Our results demonstrate that our method can detect attacks with a false positive rate of 0.052% while maintaining low computational overhead. Most importantly, it achieves a resilience rate of 99.78%, demonstrating its effectiveness in protecting IMU systems.

## 2. Related Works

This section describes existing defense methods against sensor attacks in a physical domain. Existing methods are divided into two main categories: hardware- and software-based methods.

### 2.1. Hardware-Based Methods

**Secure Hardware.** Some researchers have suggested a robust hardware design to prevent signal injection attacks on sensors [2,3,4,5]. Especially, Trippel, et al. proposed a secure low-pass filter and amplifier [5]. A secure low-pass filter can prevent injection attacks by attenuating the frequency bands of noise or malicious signals, but the disadvantages are that the frequency of movements that the sensor can measure is limited or requires many additional components. Furthermore, it was recently noted that applying a low-pass filter of 10 Hz when the resonant frequency of the sensor is in the kHz range means that this method cannot even attenuate false data caused by malicious signal injection [9]. Likewise, a secure amplifier ensures that no clipping occurs and, thus, the sensor data are not distorted. However, this increases both cost and size because more chip components and circuitry are required for the sensor.

**Shielding.** Shielding is a method of physically isolating the sensor from malicious acoustic signal injection (e.g., by covering the IMU sensor with an additional case) [2,3,4,5].

**Dummy/Redundant Sensor.** In addition, there are redundancy-based methods that can detect and respond to malicious signals by applying additional sensors [2,3,4]. For example, if an attacker injects acoustic signals into an IMU sensor, these can be detected by placing an additional microphone near the sensor.

Unfortunately, all hardware-based methods are inevitably limited in practical application on currently deployed devices.

### 2.2. Software-Based Methods

**Dynamic Sampling.** Dynamic sampling methods include random sampling and out-of-phase sampling [4,5]. Random sampling involves sampling the sensor data over a random period, and out-of-phase sampling samples data at a 180^∘^-phase delay. Software-based methods merely change the shape of the waveform that the attacker can inject; they are not solutions to the vulnerability itself. Thus, they are not sufficient countermeasures when deployed alone [10]. Recently, the method has been studied to provide resilience against signal injection attacks by sampling jitter as noise [11]. However, this method has a 30 ms time delay between the board and the server.

**Sensor Fusion.** Many claim that sensor fusion can make signal injection attacks difficult because this method fuses data measured from different sensors [3,4,6]. However, Nashimoto et al. recently demonstrated that a Kalman Filter algorithm could be vulnerable against such attacks [7]. Tharayil et al. also proposed a sensor fusion method based on a mathematical relation between gyroscope and magnetometer data, but it only detects signal injection attacks [6]. On the other hand, our method estimates one sensor’s data from the other’s data, and based on this, it can both detect the attack and provide resilience. In other words, even in the event of a signal injection attack, the device can behave in a near-normal state thanks to the estimated data. Tharayil et al. did propose a detection method for a single sensor based on machine learning [6], but this method requires a pre-learning process, which is a slight disadvantage, and resilience is ultimately still not conclusively proven.

**System Model.** There is one new method that detects attacks and provides resilience based on a system model of the sensor [8]. The method first constructs a system model of the behavior of the controller, actuators, and vehicle physics and dynamics, and then it predicts the next physical state, given the system input (i.e., reference) and the current state. Since this method is based on the system model of the sensor, it needs accurate modeling (e.g., parameters of the model). Also, this method can temporarily prevent sensor attacks, but the resilience duration is only 10 s in most cases. In other words, it can resist an attack lasting for 10 s, but resilience cannot be guaranteed for attacks lasting longer than that duration. Moreover, it was recently demonstrated that acoustic signal injection attacks on gyroscopes are possible for long periods of time (e.g., a range of up to one minute), but providing only 10 s of resilience is not enough. Additionally, another method has been studied to restore the compromised attitude states using the system model and the remaining position and heading information [12]. However, this method is also inadequate for extended attacks, as it provides attitude stabilization for only 16 s. In contrast, our proposed method is resilient to longer attack durations. For example, for a 61-second-long piece of data (measured with a sensor located on the right lower arm while the participant was running) in one of the open datasets, MHEALTH, the path tracked from the estimated data and that from the actual measured data return an average correlation of 0.9997. For a 223-second-long piece of data (measured with a sensor located on the hand as the participant was running) in the open dataset, PAMAP2, the average path correlation is 0.9575. Accordingly, our method can provide long-term resilience. There are other methods of detecting attacks by estimating system state, but these also depend on the accuracy of the system model [13,14,15]. There is also a study for a method called RVPLAYER, which identifies the root cause of an accident based on the RV forensic system [16]. However, this method does not provide resilience either.

Table 1 summarizes the key differences between existing methods and our approach.

## 3. Background

This section describes the fundamental background knowledge behind the construction of the mathematical models we propose in this paper, including the sensor’s coordinate system and measurement principle. It also details existing methods that handle signal injection attacks on IMU sensors.

### 3.1. Coordinate System

A sensor measures a physical quantity and converts it into a signal that can be read by an observer or device. Here, there are typically two coordinate frames that analyze the measured physical quantity: a body frame and an inertial frame.

**Body Frame.** A body frame is defined as the coordinate system of the object in which the sensor is embedded (i.e., a coordinate system that coincides with the axis of the measuring sensor). For example, when a smartphone is placed on a level tabletop, the x-axis points to the right, the z-axis points up, and the y-axis points to the front. Here, the IMU sensor measures the physical quantity with respect to the body frame.

**Figure 1:**
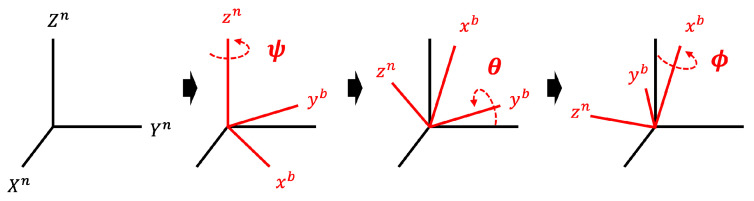
Three rotations of body frame *b* sequentially in the order of *z*, *y*, and *x* (i.e., ψ, θ, and ϕ).

**Inertial Frame.** An inertial frame is an Earth-fixed coordinate system. The coordinates can be based on cardinal directions or arbitrary directions within the room floor plan. Changing the data measured relative to the body frame *b* into data relative to the inertial frame *n* facilitates a more intuitive and standardized analysis of object movement, as it provides a consistent reference independent of the sensor’s orientation [17]. We denote unit vectors corresponding to the inertial frame and the body frame as ${\left[X,Y,Z\right]}^{n}$ and ${\left[x,y,z\right]}^{b}$, respectively. A useful way to convert one vector to another is to use matrix multiplication. The matrix for transformation is called the direction cosine matrix (DCM), or the rotation matrix, denoted by *R*. Note that it has a property that transposes matrix $R^{T}$ to be equal to the inverse $R^{-1}$ (i.e., $R^{T}=R^{-1}$) because this matrix consists of orthogonal unit vectors. As shown in Figure 1, we use a common standard method to perform a sequential transformation of rotations in the order of $z^{b}$, $y^{b}$, and $x^{b}$ (i.e., Body 3-2-1). In other words, ${\left[X,Y,Z\right]}^{n}$ vector is first multiplied by ψ rotation, then θ rotation, and finally the result is multiplied by ϕ rotation matrix. Note that rotations of ϕ, θ, and ψ refer to rotations around the x-axis, y-axis, and z-axis of the body frame, respectively. Consequently, the rotation matrix *R* is expressed as:

(1) $$\left[\begin{array}{ccc} 1 & 0 & 0\\ 0 & cos(\phi ) & sin(\phi )\\ 0 & -sin(\phi ) & cos(\phi ) \end{array}\right]\left[\begin{array}{ccc} cos(\theta ) & 0 & -sin(\theta )\\ 0 & 1 & 0\\ sin(\theta ) & 0 & cos(\theta ) \end{array}\right]\left[\begin{array}{ccc} cos(\psi ) & sin(\psi ) & 0\\ -sin(\psi ) & cos(\psi ) & 0\\ 0 & 0 & 1 \end{array}\right]$$

Note that the three rotation matrices of Equation (1) can be simplified into a single matrix that transforms inertial frame *n* to body frame *b*. As a result, the equation after performing the rotation in reverse from body frame *b* to inertial frame *n* is:

(2) $${\left[\begin{array}{c} X\\ Y\\ Z \end{array}\right]}^{n}=R^{-1}{\left[\begin{array}{c} x\\ y\\ z \end{array}\right]}^{b}=R^{T}{\left[\begin{array}{c} x\\ y\\ z \end{array}\right]}^{b}$$

### 3.2. IMU Sensor Types

**Gyroscope.** A gyroscope measures the three-axis angular velocity ${\left[\omega_{x},\omega_{y},\omega_{z}\right]}^{b}$ relative to body frame *b* via the Coriolis effect. In physics, the Coriolis force is an inertial force that acts upon an object moving within the body frame as it rotates with respect to the inertial frame. Here, the deflection of the object due to the Coriolis force is called the Coriolis effect. Typically, gyroscopes use a tuning fork configuration, in which two masses are connected by a spring. When an angular velocity is applied, the Coriolis force on each mass acts in the opposite direction, and consequently, the change in capacitance is directly proportional to the angular velocity. On the other hand, when linear acceleration is applied, the two masses move in the same direction, resulting in no change in capacitance and a measured angular velocity of zero.

**Magnetometer.** A magnetometer measures the strength and direction of the magnetic field relative to body frame *b*. Here, the three-axis components ${\left[M_{x},M_{y},M_{z}\right]}^{b}$ output by the magnetometer represent the vector tangent to the magnetic field line at the sensor’s current position. At the Earth’s magnetic poles, the magnetic field line and the Earth’s horizontal planes are perpendicular (i.e., the angle of inclination is 90^∘^), whereas at the equator, they are horizontal and the angle of inclination is 0^∘^. Typically, to measure the surrounding magnetic field, the magnetometer relies on the magneto-resistance of a permalloy, which changes with shifts in the magnetic field. Since magnetometers generally complete the full range of their three-dimensional rotation, ideal magnetic data should form a perfect sphere around the origin, assuming that the magnetic field is undisturbed. However, in most practical applications, magnetometer data are distorted by the magnetic fields generated either on the sensor circuit board or in the surrounding environment. In general, the distortions are classified as hard iron or soft iron. Hard iron distortion is caused by an object that creates a magnetic field. For example, when a piece of magnetic material is physically attached to a sensor, a hard iron distortion occurs and a permanent bias is created in the sensor output. This distortion moves the center of the sphere away from the origin. Soft iron distortion, on the other hand, stretches or distorts the existing magnetic field and is commonly caused by metals such as nickel and iron. This distortion warps the sphere into an ellipsoid.

To mitigate these distortions, a calibration technique is applied to correct both hard iron and soft iron effects. Hard iron distortions are compensated by subtracting the mean offset from the raw magnetometer readings, effectively shifting the distorted sphere back to the origin. Soft iron distortions require an additional transformation using a correction matrix that reshapes the distorted ellipsoid into a sphere. This matrix is computed by analyzing the distribution of the measured magnetic field values and determining the necessary scaling and rotation adjustments. To ensure accurate calibration, an optimization-based approach iteratively refines the transformation parameters. This method estimates the optimal bias vector and correction matrix by minimizing the error between the measured data and an ideal spherical distribution. The calibration process consists of two steps: first, the initial transformation parameters are determined using least-squares ellipsoid fitting; second, a refinement step dynamically optimizes the calibration parameters to further reduce residual distortions. This approach enhances the accuracy of magnetometer readings and improves robustness against environmental disturbances.

### 3.3. Signal Injection Attacks

Signal injection attacks exploit physical vulnerabilities in sensor systems by injecting malicious signals that manipulate sensor outputs. Unlike software-based attacks, these threats interfere directly with sensor hardware, bypassing traditional security mechanisms such as encryption and authentication.

It is widely known that the gyroscope is sensitive to acoustic signals with its resonant frequency. Recently, Khazaaleh et al. showed that misalignment between the sensing axis and the drive axis of the gyroscope is the main cause of acoustic vulnerability [9]. Many researchers have explored these vulnerabilities through attacks, and Son et al. conducted the first attack study on a drone [2]. The initial attempt was a simple attack that injected acoustic signals into the built-in gyroscope to make the drone’s movements uncontrollable. A speaker generated a sound pressure level (SPL) of up to 113 dB at the resonant frequency, and the attack distance was as short as 10 cm. Since then, Wang et al. demonstrated signal injection attacks against gyroscopes in a virtual reality headset and a self-driving car [3]. Recently, Tu et al. showed that the gyroscope can be controlled in a much more granular way [4]; they demonstrated that long-period (up to the one-minute range) and long-range (up to 7.8 m at a maximum SPL of 135 dB) attacks were possible when targeting built-in gyroscopes of a phone, a scooter, a stabilizer, a screwdriver, a VR headset, and other devices.

These studies have significantly enhanced our understanding of signal injection attacks, but defending against such threats remains a major challenge. Therefore, we focus on detecting and responding to attacks that exploit vulnerabilities at the physical layer, beyond traditional cybersecurity defenses.

## 4. Attack Model

An attacker can interfere with the normal functioning of a device or make it behave differently by injecting malicious signals into the IMU sensor. We propose a system that is resilient against these signal injection attacks. For example, if a device equipped with an IMU sensor is a drone or a self-driving car, our goal is to ensure that it follows a pattern similar to its original intention or function even if malicious signals are injected.

We assume an attacker who maliciously injects acoustic signals into the gyroscope sensor at the physical layer. The attacker aims to introduce a perturbation δω into the gyroscope’s measured angular velocity $\omega^{b}$, such that the compromised measurement ${\tilde{\omega }}^{b}$ deviates from the true value:

(3) $${\tilde{\omega }}^{b}=\omega^{b}+\delta \omega .$$

The injected perturbation δω depends on the attack signal s(t), which is typically designed to match the gyroscope’s resonant frequency $f_{r}$:

(4) $$\delta \omega =A\sin(2\pi f_{r}t+\phi ),$$

where *A* is the amplitude of the injected signal, $f_{r}$ is the gyroscope’s resonant frequency, and ϕ is the phase shift.

The attacker is assumed to inject the signal from as far a distance as possible to maximize both the feasibility and detectability of the attack. For instance, it has been demonstrated that acoustic signal injection attacks on gyroscopes can be effective from a long distance, such as 7.8 m away [4]. Additionally, we assume the attacker cannot physically tamper with the target device or modify its software in any way. The term “device” in this paper refers to any equipment equipped with an IMU sensor, such as drones or ground robots.

**Exclusion of Magnetic Signal Injection Attacks.** While magnetic signal injection attacks on magnetometers have been explored in prior research, we exclude them from our study due to their limited real-world feasibility. These attacks require the target device to be in close physical proximity (within approximately 5 cm of the attacker), making them impractical for dynamic environments such as drones, mobile systems, and autonomous robots [6,7]. Unlike acoustic signal injection attacks on gyroscopes, which can be conducted from several meters away, magnetometer attacks require extremely close-range access, limiting their practicality in real-world adversarial scenarios.

Given these constraints, our study focuses on acoustic signal injection attacks on gyroscopes, which have been demonstrated to be highly effective even from a long distance. This choice aligns with our goal of developing a resilient IMU system against real-world, long-range adversarial threats.

## 5. Our Method

This section presents our proposed method of a resilient IMU system method against signal injection attacks based on a mathematical model.

### 5.1. Estimation Model

The magnetometer outputs a magnetic field vector $M^{b}\left(t\right)=[M_{x}\left(t\right),$ $M_{y}\left(t\right),M_{z}{\left(t\right)]}^{b}$ tangent to the magnetic field line at time *t*. In other words, even if the magnetometer rotates, it outputs a magnetic field vector that is always tangential to the magnetic field line at the current location of the sensor. Accordingly, the gyroscope data represents the change rate of the angle of the magnetic field vector with respect to each axis of body frame *b*. Based on this fact, some researchers did propose a way to estimate gyroscope data from magnetometer data [18], but the estimation method in this study is incomplete. As such, we propose a different estimation method in this paper.

**Figure 2:**
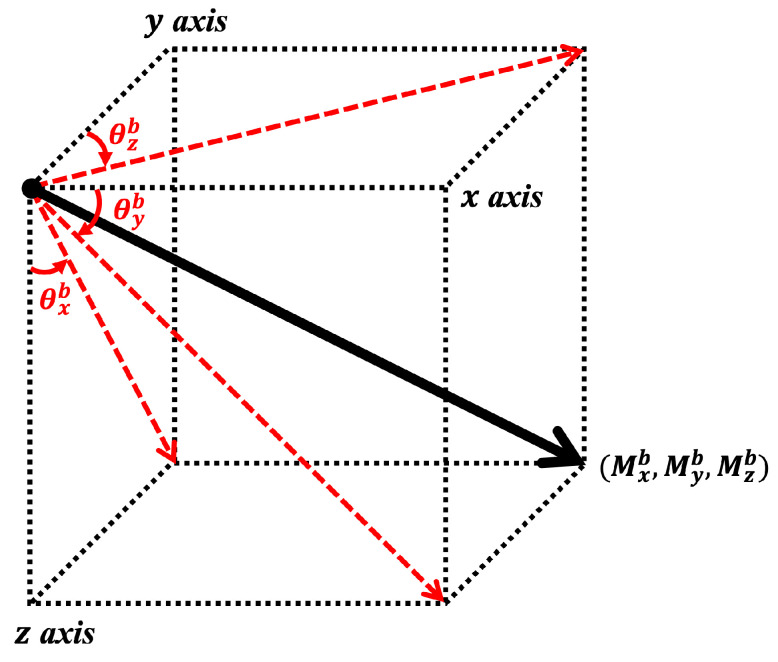
Angles $\theta^{b}$ formed by magnetometer data with respect to body frame *b*.

As shown in Figure 2, angles $\theta^{b}\left(t\right)={\left[\theta_{x}\left(t\right),\theta_{y}\left(t\right),\theta_{z}\left(t\right)\right]}^{b}$ formed by magnetic field vector $M^{b}\left(t\right)$ with respect to each axis of body frame *b* at time *t* become:

(5) $$\theta_{x}^{b}\left(t\right)=\arctan\frac{M_{y}^{b}\left(t\right)}{M_{z}^{b}\left(t\right)}$$

(6) $$\theta_{y}^{b}\left(t\right)=\arctan\frac{M_{z}^{b}\left(t\right)}{M_{x}^{b}\left(t\right)}$$

(7) $$\theta_{z}^{b}\left(t\right)=\arctan\frac{M_{x}^{b}\left(t\right)}{M_{y}^{b}\left(t\right)}$$

As a result, gyroscope data are estimated as the first derivative of angle $\theta^{b}\left(t\right)$ with respect to time *t*. The estimated gyroscope data ${\hat{\omega }}^{b}\left(t\right)$ are computed as:

(8) $${\hat{\omega }}_{x}^{b}\left(t\right)=\frac{\frac{d}{dt}M_{y}^{b}\left(t\right)\cdot M_{z}^{b}\left(t\right)-M_{y}^{b}\left(t\right)\cdot \frac{d}{dt}M_{z}^{b}\left(t\right)}{{\left(M_{y}^{b}\left(t\right)\right)}^{2}+{\left(M_{z}^{b}\left(t\right)\right)}^{2}}$$

(9) $${\hat{\omega }}_{y}^{b}\left(t\right)=\frac{M_{x}^{b}\left(t\right)\cdot \frac{d}{dt}M_{z}^{b}\left(t\right)-\frac{d}{dt}M_{x}^{b}\left(t\right)\cdot M_{z}^{b}\left(t\right)}{{\left(M_{x}^{b}\left(t\right)\right)}^{2}+{\left(M_{z}^{b}\left(t\right)\right)}^{2}}$$

(10) $${\hat{\omega }}_{z}^{b}\left(t\right)=\frac{\frac{d}{dt}M_{x}^{b}\left(t\right)\cdot M_{y}^{b}\left(t\right)-M_{x}^{b}\left(t\right)\cdot \frac{d}{dt}M_{y}^{b}\left(t\right)}{{\left(M_{x}^{b}\left(t\right)\right)}^{2}+{\left(M_{y}^{b}\left(t\right)\right)}^{2}}$$

Because of the discrete sensor measurements, the continuous derivative cannot be used, so the derivative value at time *t* should be approximated using a differential quotient—the average rate of change of the function over the interval. In this paper, we use the central difference to approximate derivatives because this provides us with the closest approximation [19]. That is, the discrete derivative at time *t* is calculated by averaging the difference between measurements at times t−1 and t+1 and then dividing it by the actual elapsed time between these measurements. Accordingly, for sampling rate *f*, the first discrete derivative of $M^{b}\left(t\right)$ at time *t* can be expressed as $\frac{d}{dt}M^{b}\left(t\right)=\frac{f}{2}\cdot \left(M^{b}\left(t+1\right)-M^{b}\left(t-1\right)\right)$.

**Figure 3:**
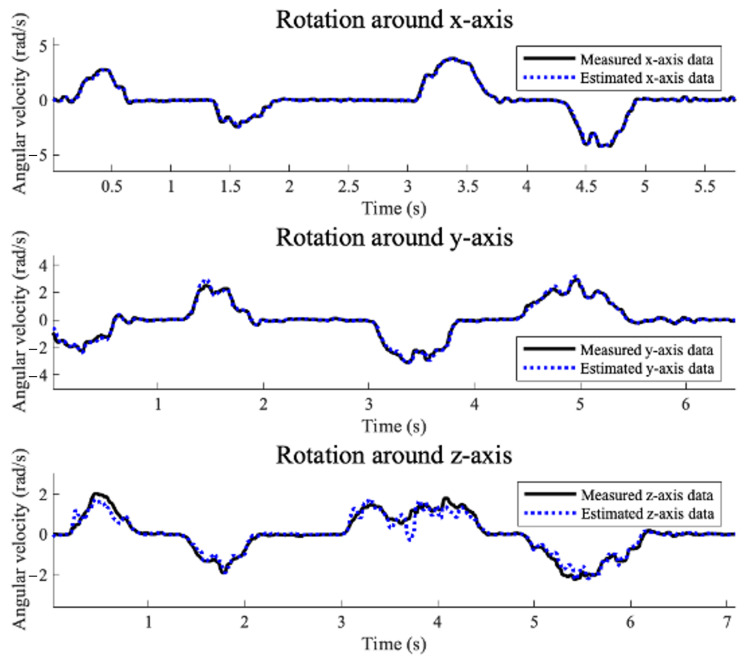
Measured and estimated gyroscope data when the iPhone 13 Pro Max rotates about the x-axis, y-axis, and z-axis relative to the body frame.

Figure 3 shows measured and estimated gyroscope data with respect to the body frame when the iPhone 13 Pro Max is rotated around each axis. The average errors in the norm of the difference between the measured and estimated three-axis gyroscope data for each rotation are 0.0565 (rad/s), 0.0788 (rad/s), and 0.1003 (rad/s), respectively.

### 5.2. Attack Resilience

**Figure 4:**
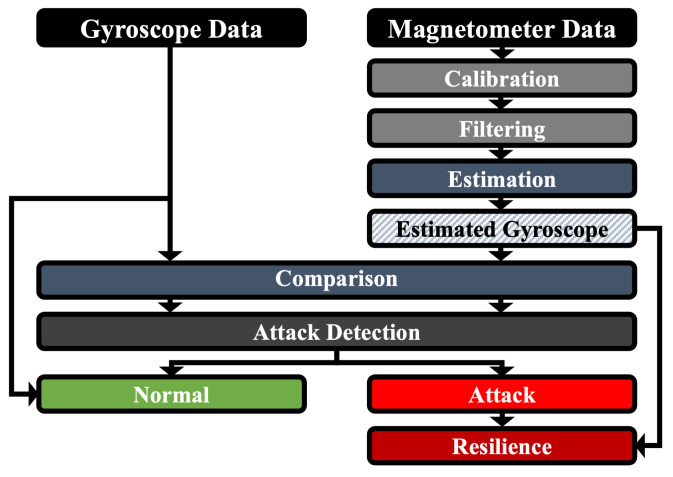
Overview of our proposed attack-resilient IMU system.

The overall process of our proposed method is shown in Figure 4. When an attack occurs, the relation between gyroscope data and magnetometer data is broken. If the gyroscope is deemed to be under attack, gyroscope data are then estimated from the magnetometer data using Equations (8)–(10). The estimated gyroscope data are then compared with the actual gyroscope data measurements based on a mean absolute error within window *N*. Regarding estimated gyroscope data ${\hat{\omega }}^{b}$ and measured gyroscope data $\omega^{b}$, the gyroscope estimation error (GEE) is calculated as $\frac{1}{N}\sum_{k=1}^{N}∥{\hat{\omega }}_{k}^{b}-\omega_{k}^{b}∥$. If the GEE is greater than a certain threshold, we can determine that an acoustic signal injection attack has occurred on the gyroscope. At this juncture, the estimated gyroscope data are used instead of the actual gyroscope data to facilitate behavior that is similar to normal functioning. Afterward, if the GEE falls below the threshold for a set period of time, we can determine that the attack has stopped, and the actual measured gyroscope data are used again.

Algorithm 1 presents the step-by-step procedure of our attack resilience mechanism. This algorithm describes how the system estimates gyroscope data from the magnetometer, detects an attack, and switches to estimated data when necessary. The algorithm continuously monitors the gyroscope estimation error (GEE) and determines when to revert to actual gyroscope measurements based on a predefined threshold.
**Algorithm 1** Attack-Resilient IMU System1:**Input:** Gyroscope data $\omega^{b}$, magnetometer data $M^{b}$, window size *N*, threshold τ2:**Output:** Estimated gyroscope data ${\hat{\omega }}^{b}$3:Initialize estimated gyroscope data ${\hat{\omega }}^{b}=0$4:**for** each time step *t* **do**5:    Compute estimated gyroscope data using magnetometer:6:       ${\hat{\omega }}_{x}^{b}\left(t\right)=\frac{\frac{d}{dt}M_{y}^{b}\left(t\right)\cdot M_{z}^{b}\left(t\right)-M_{y}^{b}\left(t\right)\cdot \frac{d}{dt}M_{z}^{b}\left(t\right)}{{\left(M_{y}^{b}\left(t\right)\right)}^{2}+{\left(M_{z}^{b}\left(t\right)\right)}^{2}}$7:       (Similarly for ${\hat{\omega }}_{y}^{b}\left(t\right)$ and ${\hat{\omega }}_{z}^{b}\left(t\right)$)8:    Compute GEE (Gyroscope Estimation Error):9:       $GEE=\frac{1}{N}\sum_{k=1}^{N}∥{\hat{\omega }}_{k}^{b}-\omega_{k}^{b}∥$10:    **if** GEE>τ **then**11:        **Attack detected:** Use estimated gyroscope data12:           $\omega^{b}={\hat{\omega }}^{b}$13:    **else**14:        Use actual gyroscope data15:    **end if**16:**end for**17:**Return** $\omega^{b}$

**Figure 5:**
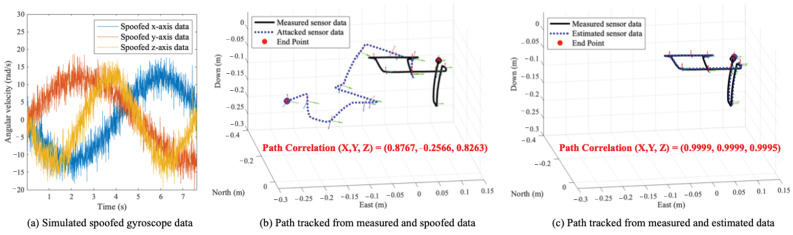
Simulated spoofed gyroscope data and a comparison of paths from measured data, attacked data, and estimated data.

**Simple Evaluation** We measure the IMU sensor data of a commercial smartphone, an iPhone 13 Pro Max, while it is moving across the circumference of a square, which is about 0.1 m, and then while it is moving down 0.1 m and up 0.1 m again. The movement of the iPhone 13 Pro Max is shown in black solid lines in Figure 5b,c. We simulate spoofed gyroscope data as shown in Figure 5a; here, the average error of the norm between the measured gyroscope data and the spoofed gyroscope data is 8.1407 (rad/s). The path tracked from the spoofed gyroscope data is represented by blue dotted lines in Figure 5b. As a result, the correlation between the coordinates of the paths tracked from the actual measured data and the attacked data is (0.8767, −0.2566, 0.8263), and the average of the three correlation values is 0.4821, as shown in Figure 5b. In other words, the path after the gyroscope is spoofed and has low similarity with the actual path. On the other hand, the blue dotted lines in Figure 5c show the the path tracked from the data estimated by our proposed method. As shown in Figure 5c, the correlation between the path tracked from the actual data and the path tracked from the estimated data is (0.9999, 0.9999, 0.9995), which is very high.

## 6. Experimental Setup

The following details the evaluation of our proposed IMU system that is resilient against signal injection attacks.

**Sensor Types.** We gather data to ascertain whether acoustic signals that detrimentally affect the gyroscopes also affect the magnetometers, and also if the magnetometer signals affect the gyroscope signals in reverse. Accordingly, we use a total of ten IMU sensor models: MPU-9250 [20], MPU-9150 [21], HMC5883L [22], GY-85 [23], MAG3110 [24], LIS3MDL [25], ASD2511-R-C [26], ASD2511-R-N [27], LSM303DLHC [28], and MLX90393 [29]. The MPU-9250, MPU-9150, GY-85, and ASD2511-R-N models are nine-axis sensors consisting of accelerometers, gyroscopes, and magnetometers; the HMC5883L, MAG3110, LIS3MDL, ASD2511-R-C, and MLX90393 models are three-axis magnetometers; and the LSM303DLHC is a six-axis sensor consisting of an accelerometer and a magnetometer. In addition, we utilize the Keysight N9310A RF signal generator [30] to generate acoustic signals between the frequency range of 19 kHz to 30 kHz, and an ultrasonic speaker [31] and an amplifier [32] are used to emit the generated acoustic signals.

**Simulator.** We use a simulator (i.e., ardupilot) to measure sensor data when a drone is flying dynamically. It makes 25 flights under normal condition and 25 flights under attack condition.

**Figure 6:**
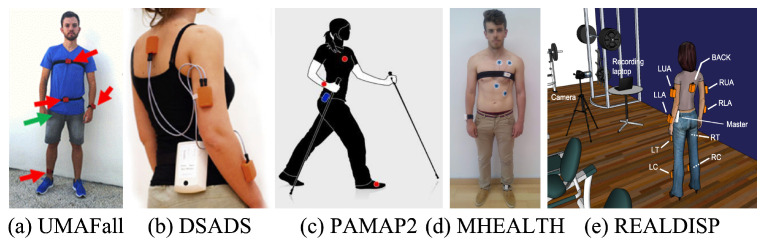
Experimental environment of open datasets used in our evaluation (source: (**a**) UMAFall [33], (**b**) DSADS [36], (**c**) PAMAP2 [41], (**d**) MHEALTH [42], and (**e**) REALDISP [43]).

**Open Datasets.** We use five open datasets to evaluate whether the estimated sensor data are similar to the actual measured data. Each open dataset contains a large amount of IMU data while multiple participants performed various physical activities. We would like to mention that a set of publicly available datasets is able to show a clear and unbiased evaluation result. Figure 6 shows the experimental environment for measuring participant movements for each open dataset. A summary of the open datasets we used for evaluation is given in Table 2.

The first open dataset is UMAFall, which contains mobility traces generated by 19 participants who performed predetermined activities of daily life (ADLs) and falls on a mattress [33,34,35]. The activities include nine types of ADL and three types of falls. The ADL types are walking, jogging, body bending, hopping, climbing stairs (up/down), lying down in and getting up from a bed, sitting down (and getting up) on (from) a chair. Fall types are lateral, frontal, and backward. As all participants performed each activity, the Samsung S5 and LG G4 smartphones (located in a pant pocket as shown in Figure 6a) measured the IMU data using built-in sensors at a sampling rate of 200 Hz. All of these experiments were conducted in a home environment. UMAFall also has data measured with SensorTag kits (located in red arrows), but we do not use these data for evaluation because the sampling rate of 20 Hz is too low.

The Daily and Sports Activities Data Set (DSADS) contains IMU sensor data taken while eight participants performed 19 different physical activities [36,37,38,39]. The activity types are sitting/standing, lying on back/right side, ascending/descending stairs, standing still/moving around in an elevator, walking in a parking lot, walking on a treadmill (at a speed of 4 km/h with no incline/at a speed of 4 km/h with an incline of 15^∘^), running on a treadmill at a speed of 8 km/h, exercising on a stair stepper/cross trainer, riding an exercise bike while seated/standing up, rowing, jumping, and playing basketball. While each participant performed the activities, five MTx 3-DOF orientation trackers [40] (located on the torso, right arm, left arm, right leg, and left leg as shown in Figure 6b) measured the IMU data at a sampling rate of 25 Hz. All activities were performed inside the Bilkent University Sports Hall building and on a flat outdoor space on the campus.

The Physical Activity Monitoring (PAMAP2) dataset contains sensor data taken while nine participants performed 18 different physical activities [41,44,45]. The activity types are lying down, sitting/standing, walking/running, cycling, Nordic walking, watching TV, working on a computer, driving a car, ascending/descending stairs, vacuuming, ironing, folding laundry, cleaning around the house, playing soccer, and jumping rope. While each participant performed the activities, three Colibri wireless sensors [46] (located on the wrist of the dominant arm, chest, and ankle of the dominant leg as shown in Figure 6c) are used to measure the IMU data at a sampling rate of 100 Hz. Information about the detailed locations for measuring data is unknown.

The Mobile HEALTH (MHEALTH) dataset contains sensor data taken while ten participants performed 12 types of body motions [42,47,48]. The activity types are standing still, sitting, relaxing while lying down, walking, climbing stairs, bending forward at the waist, lifting the arms, bending the knees (crouching), cycling, jogging, running, and jumping forward/backward. While each participant performed the activities, two Shimmer2 sensors [49] (located on the left ankle and right lower arm as shown in Figure 6d) were used to measure the IMU data at a sampling rate of 50 Hz. Note that these data were collected in an environment outside laboratory conditions where there were no restrictions on participant movements, and information about the detailed locations for measuring data is unknown.

Finally, the REAListic sensor DISPlacement (REALDISP) dataset contains sensor data measured while 17 participants performed 33 different physical activities [43,50,51]. The activity types are walking, jogging, running, jumping (up, forward/backward, sideways, with legs open/arms closed, and with a rope), twisting at the trunk, bending at the waist and rotating, stretching the heels backward, laterally bending and elevating the arms, stretching forward repeatedly, twisting the upper trunk and lower body in opposite directions, holding the arms out in front of the body, clapping hands, crossing arms, rotating shoulders high/low, rotating arms inward, bringing knees up to the chest/bending, touching heels to the rear, rotating the knees, rowing, riding an elliptical bike, and cycling. While each participant performed the corresponding activities, nine Xsens MTx sensors [40] (located on the right lower arm, right upper arm, back, left upper arm, left lower arm, right calf, right thigh, left thigh, and left calf as shown in Figure 6e) are used to measure the IMU data at a sampling rate of 50 Hz. All activities were performed in a physical fitness room.

**Data Processing.** There were several missing values in some open datasets due to wireless network delays during transmission. These were marked as “Nan”. For our research, all datasets underwent a pre-processing process to remove all Nan values. Thereafter, the effects of hard iron and soft iron distortion on the magnetometer data were effectively removed during calibration. We remove noise from the gyroscope data and the magnetometer data with a moving median filter based on a fixed window. The gyroscope data are then estimated based on methods mentioned in Section 5.1.

**Path Tracking Method.** To evaluate our attack-resilient IMU system, we analyze whether the path tracked from the actual measured data and the path tracked from the data estimated by our method follow a similar pattern. We use a method of tracking a device’s path using the IMU sensor data. Here, the accelerometer data are used only as a reference for the tracking method to evaluate our method. Since the resonant frequencies of accelerometers are usually within the range of human hearing, and those of gyroscopes are often in the ultrasonic band [4], malicious acoustic signals that affect gyroscope data do not affect accelerometer data. To track the path, we first detect stationary periods in the accelerometer data. We compute the magnitude of the accelerometer data and apply a high-pass filter with a cutoff frequency of 0.001 Hz. Then, absolute values of the filtered data are computed. Since all open datasets used for our evaluation are measurements of human activities, and the maximum frequency of human movements is 20 Hz [52], a low-pass filter with a cut-off frequency of 20 Hz is applied to the absolute values. Here, according to the Nyquist theorem, if the sampling rate is greater than 40 Hz, a low-pass filter is applied; otherwise, it is not applied. Note that the cut-off frequency may be different according to the types of datasets. Consequently, if the final obtained value is less than a threshold of 0.05, it is deemed a stationary period. Thereafter, quaternion angles (a convenient mathematical notation for representing spatial orientations and rotations of elements in three-dimensional spaces [53]) are computed using the sensor data through the AHRS algorithm. Based on the obtained quaternian angles, the accelerometer data (i.e., with respect to body frame *b*) are rotated to the data with respect to inertial frame *n*. The accelerometer data are integrated to obtain velocity values, and integral biases during non-stationary periods are also removed. Finally, positions are obtained by integrating the velocity values, and the device’s path can be tracked based on the obtained position values.

## 7. Evaluation Metric

We define thresholds for the GEE to detect signal injection attacks. According to three types of attacks performed by Tu et al. [4], we set the threshold for the GEE to 0.25 (rad/s), which is the smallest norm of the difference between the normal gyroscope data. We use a percentage of the data whose estimation error between the actual measured and estimated data is greater than each threshold as a performance metric for the false positive rate of attack detection.

We evaluate our method based on whether the device can follow a pattern similar to its original intention or function even if a signal injection attack occurred on the device. Accordingly, we use the Pearson correlation coefficient between the path tracked from the actual measured data (i.e., the actual path) and the path tracked from the estimated data (i.e., the estimated path). The closer the correlation values are to 1, the closer the estimated path is to the actual path. This means that the IMU system is resilient even if it comes under attack. For both the actual path $p=[p_{x},p_{y},p_{z}]$ and the estimated path $\hat{p}=\left[{\hat{p}}_{x},{\hat{p}}_{y},{\hat{p}}_{z}\right]$ with $∥p∥=∥\hat{p}∥=L$, the path correlation value $P_{corr,i}$ for each coordinate *i* is defined as:

(11) $$P_{corr,i}=\frac{\sum_{k=1}^{L}\left(p_{i,k}-\bar{p_{i}}\right)\left({\hat{p}}_{i,k}-\bar{{\hat{p}}_{i}}\right)}{\sqrt{\sum_{k=1}^{L}{\left(p_{i,k}-\bar{p_{i}}\right)}^{2}}\sqrt{\sum_{k=1}^{L}{\left({\hat{p}}_{i,k}-\bar{{\hat{p}}_{i}}\right)}^{2}}}$$

For convenience in analyzing, we define path correlation $P_{corr}$ as the average of the path correlation values for the x, y, and z coordinates.

## 8. Evaluation

We evaluate our attack-resilient IMU system using five open datasets that measure various movements with IMU sensors. The results show that across all five open datasets, our method can detect signal injection attacks on IMU sensors with a false positive rate of 0.052% and has a resilience of 99.78% against attacks.

### 8.1. Interference Experiments

We gather data to ascertain whether acoustic signals that detrimentally affect the gyroscopes also affect the magnetometers. We use a total of ten IMU sensor models. The acoustic signals are injected at a distance of 5 cm into magnetometers while the frequency of the signals increases stepwise by 0.1 kHz from 19 kHz to 30 kHz. Note that, in general, the frequency at which the gyroscope has an effect is between 19 kHz and 30 kHz [2]. As a result, none of the magnetometers respond to acoustic signals with a frequency range of 19 kHz to 30 kHz. In other words, an acoustic signal injection attack only affects the gyroscope.

### 8.2. Simulation-Based Results

When the drone is flying dynamically in the simulation, our method is able to properly estimate sensor data. Under normal conditions, the average error between estimated data and actual data is 0.0141 (rad/s) for the gyroscope. In other words, the average GEE is below the determined threshold. Also, the average correlation $P_{corr}$ between the path when an attack occurred and the path in an actual normal condition comes out as 0.0641 (i.e., mission failure), but when our resilience method is applied, it comes out as 0.9071.

### 8.3. Evaluation Using Open Datasets

We also evaluate our method with open datasets which contains a large amount of IMU data while multiple participants performed various physical activities.

**Sensor Estimation Error.** Using data for each type of physical activity in the open datasets, we estimate gyroscope data from actual measured magnetometer data. We then calculate a GEE. The average GEE of the total data in UMAFall (#1) is 0.0016 (rad/s), and the maximum GEE is 0.0137 (rad/s). For DSADS (#2), the average GEE is 0.0054 (rad/s) and the maximum GEE is 0.0494 (rad/s). The average GEE of PAMAP2 (#3) is 0.0395 (rad/s), and the maximum GEE is 0.1995 (rad/s). For MHEALTH (#4), the average GEE is 0.0020 (rad/s), and the maximum GEE is 0.0104 (rad/s). Lastly, the average GEE of REALDISP (#5) is 0.0497 (rad/s), and the maximum is 0.3547 (rad/s).

Based on the threshold for a GEE of 0.25 (rad/s), the GEEs of all open datasets except REALDISP (#5)—the last open dataset—are below the threshold. For the REALDISP (#5), data with GEEs greater than or equal to 0.25 (rad/s) and less than 0.3 (rad/s) are 0.261% (i.e., 23/8820), while data with GEEs greater than or equal to 0.3 (rad/s) and less than 0.35 (rad/s) are 0.057% (i.e., 5/8820). Also, data with a GEE greater than or equal to 0.35 are 0.011% (i.e., 1/8820). As a result, when the threshold is set to 0.25 (rad/s) for a total of five open datasets, the false positive rate of detecting an acoustic signal injection attack on the gyroscope is 0.052% (i.e., 29/55,661). The results are summarized in Table 3.

**Attack Resilience.** To evaluate the performance of our resilience method in each open dataset, we compare paths tracked from the actual measured data and the estimated data through correlation. The average $P_{corr}$ of total tracked paths in UMAFall (#1) is 0.9999, and the minimum $P_{corr}$s is 0.9853. For DSADS (#2), the average $P_{corr}$ is 0.9990, and the minimum is 0.8115. The average $P_{corr}$ of PAMAP2 (#3) is 0.9813, and the minimum $P_{corr}$ is 0.7258. For MHEALTH (#4), the average $P_{corr}$ is 0.9983, and the minimum is 0.9164. Lastly, the average $P_{corr}$ of REALDISP (#5) is 0.9947, and the minimum $P_{corr}$ is 0.7156.

For the first and fourth open datasets (UMAFall (#1) and MHEALTH (#4)), all $P_{corr}$s are greater than 0.9. For the DSADS (#2), the second open dataset, data with $P_{corr}$s greater than 0.8 and less than 0.85 are 0.007% (i.e., 3/45600), while data with $P_{corr}$s greater than 0.85 and less than 0.9 are 0.031% (i.e., 14/45600). For the PAMAP2 (#3), data with $P_{corr}$s greater than 0.7 and less than 0.75 are 0.379% (i.e., 1/264), while data with $P_{corr}$s greater than 0.75 and less than 0.8 are 1.136% (i.e., 3/264). Also, data with $P_{corr}$s greater than 0.8 and less than 0.85 are 1.894% (i.e., 5/264), while data with $P_{corr}$s greater than 0.85 and less than 0.9 are 1.515% (i.e., 4/264). For the last open dataset, REALDISP (#5), data with $P_{corr}$s greater than 0.7 and less than 0.75 are 0.102% (i.e., 9/8820), while data with $P_{corr}$s greater than 0.75 and less than 0.8 are 0.170% (i.e., 15/8820). Also, the data with $P_{corr}$s greater than 0.8 and less than 0.85 are 0.272% (i.e., 24/8820), and the data with $P_{corr}$s greater than 0.85 and less than 0.9 are 0.646% (i.e., 57/8820). If the correlation between the path tracked from the actual measured data and the path tracked from the estimated data is 0.9 or greater, it is assumed that the resilient system performs well. As a result, our method is 99.78% (i.e., 55,526/55,661) resilient against attacks. Here, 0.22% of the total means that correlation $P_{corr}$ is less than 0.9, which is a very low probability value, and the lowest $P_{corr}$ is 0.7156. Therefore, even in the event that the 0.22% resilience failure occurred in the overall operation of devices, it would only deviate from the error range of the original path and would not affect overall operation. The results are summarized in Table 4.

### 8.4. Analysis on Computational Cost

**Figure 7:**
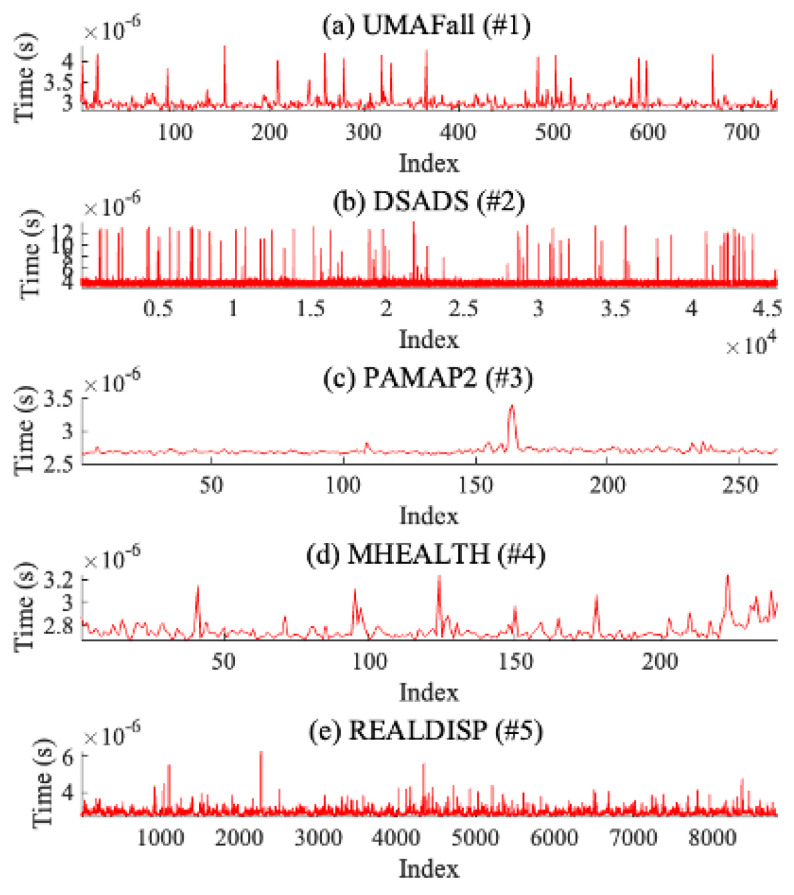
Computational time taken to estimate gyroscope data for each open dataset.

We analyze the time (i.e., latency) it takes to estimate the gyroscope data wherever this computational time may affect the real-timeness of our resilience method. From the data in each open dataset, we use Matlab to measure computational times to estimate a piece of data via our method mentioned in Section 5.1. Figure 7 shows the computational time taken to estimate gyroscope data for each open dataset. In summary, for UMAFall (#1), the average computational time is 2.979 $\times \;10^{-6}$ (s) (min: 2.798 $\times \;10^{-6}$ (s), max: 4.397 $\times \;10^{-6}$ (s)), and for DSADS (#2), the average computational time is 3.337 $\times \;10^{-6}$ (s) (min: 2.974 $\times \;10^{-6}$ (s), max: 1.382 $\times \;10^{-5}$ (s)). For PAMAP2 (#3), the average computational time is 2.6952 $\times \;10^{-6}$ (s) (min: 2.636 $\times \;10^{-6}$ (s), max: 3.398 $\times \;10^{-6}$ (s)), and for MHEALTH (#4), the average computational time is 2.752 $\times \;10^{-6}$ (s) (min: 2.667 $\times \;10^{-6}$ (s), max: 3.247 $\times \;10^{-6}$ (s)). Lastly, for REALDISP (#5), the average computational time is 2.862 $\times \;10^{-6}$ (s) (min: 2.662 $\times \;10^{-6}$ (s), max: 6.333 $\times \;10^{-6}$ (s)). In conclusion, our method of estimating gyroscope data results in lower computational costs and, therefore, can provide real-time resilience.

**Computational Overhead and Real-Time Performance.** To evaluate the impact on real-time performance, we analyze the computational complexity of our approach. Our method primarily relies on vector arithmetic and basic matrix operations, which have a computational complexity of O(1) per time step. This ensures that the computational burden does not grow with input size, making it well suited for real-time applications. In practical terms, we compare our computational cost to typical constraints in resource-constrained environments. For example, embedded systems such as IoT devices and drones often operate with processors in the range of 100 MHz to 1 GHz. Given our computational time of approximately $3\times 10^{-6}$ s per estimation, even a low-power processor (e.g., 100 MHz) can perform over 10^5^ estimations per second, which is significantly faster than the typical IMU sampling rate (100–1000 Hz). This suggests that our method introduces negligible overhead and can be executed in real time even on low-power devices. Furthermore, we analyze the memory efficiency of our method. Since it operates on a sliding window basis and does not require storing large amounts of historical data, it maintains a constant memory usage. This makes it suitable for deployment in resource-limited environments.

Our results indicate that our method introduces minimal computational overhead and is capable of running in real time, even on resource-constrained devices such as drones and IoT sensors.

### 8.5. Implementation on Devices

**Figure 8:**
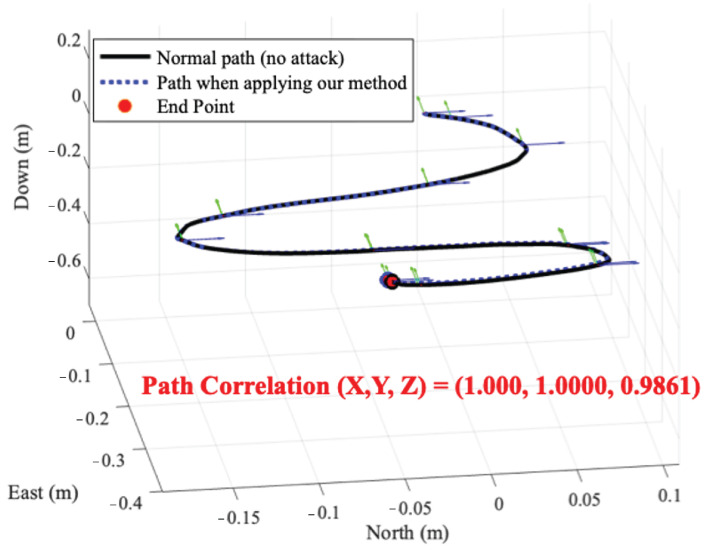
The normal driving path of the RC car and the path when our attack-resilient method is applied.

We evaluate our method when an RC car equipped with IMU sensors of the MPU-9250, MPU-9150, GY-85, and ASD2511-R-N models drives on a curved road in an indoor environment. Figure 8 shows the normal driving path of the RC car equipped with MPU-9250 and the driving path when our method is applied. The result shows that the correlation of two driving paths is (1.000, 1.0000, 0.9861). When the RC cars equipped with four IMU sensor models drive, the average correlation between the normal path and the path to which our method is applied is 0.9932. Therefore, our method shows that even if signal injection attacks occur, it is possible to make the device behave in a near-normal state.

## 9. Discussion

**Accelerometers.** In this paper, we consider signal injection attacks on gyroscopes and propose a mathematical model-based attack-resilient method for estimating gyroscope data from magnetometer data. However, many devices also contain accelerometers in addition to gyroscopes and magnetometers. An accelerometer measures the vibration or acceleration of a device’s motion. Forces caused by vibrations or changes in motion compress piezoelectric materials inside the sensor, producing a charge proportional to the applied force, and thus the acceleration. While acoustic signal injection attacks on gyroscopes exploit their resonant frequencies, accelerometers can also be manipulated through similar mechanisms. Acoustic signals at an accelerometer’s resonant frequency can displace the suspended mass inside the sensor, causing erroneous acceleration readings. Several studies have demonstrated this vulnerability [4,5,7]. For example, Trippel et al. successfully spoofed an accelerometer into outputting predefined signals, such as the word ‘WALNUT’, through acoustic interference [5]. Their attack also enabled control over remote-controlled cars and commercial fitness tracking devices. Most of these attacks were conducted at a short range (10 cm) with a speaker generating an SPL of 110 dB, and the manipulated sensor outputs lasted up to 30 s.

To detect and provide resilience against acoustic signal injection attacks on accelerometers, we expect magnetometer and gyroscope data to be useful. The frequency range of acoustic signals that affect gyroscopes typically does not interfere with accelerometers, and magnetometer readings remain unaffected by such attacks. Since magnetometer data vary based on geographic location, changes in magnetometer readings—when transformed from the body frame to the inertial frame—could potentially be used to estimate accelerometer outputs. However, this requires precise quaternion-based transformations using gyroscope data, necessitating known initial conditions such as the starting orientation. These challenges highlight the complexity of extending our approach to accelerometer-based attacks, and we plan to explore this in future work.

**Potential Extensions to Magnetometer Attacks.** Although we focus on acoustic signal injection attacks on gyroscopes in this study, magnetic signal injection attacks on magnetometers have also been explored in prior research. However, their real-world feasibility remains limited due to the short attack range (~5 cm) required for successful execution [6,7]. Future research could investigate whether novel attack techniques or environmental conditions could make magnetometer-based attacks more viable and how resilience mechanisms could be adapted accordingly.

**Real-World Deployment Considerations.** Deploying our method in real-world scenarios requires consideration of factors such as hardware variability, computational efficiency, and evolving attack conditions. While our approach is inherently robust to sensor differences, applying adaptive correction techniques could further enhance its generalizability across different devices. Given its low computational complexity of O(1) per time step, our method is suitable for resource-constrained devices, though further optimization in memory management and hardware acceleration could improve efficiency. Additionally, optimizing detection threshold could enhance resilience against extremely prolonged attacks. These considerations highlight potential avenues for refining our approach and ensuring broader applicability in real-world environments.

Another important consideration is the ability to predict and mitigate sensor anomalies induced by signal injection attacks before they cause system-level failures. While our approach focuses on real-time resilience, incorporating fault predictability mechanisms could further improve robustness. Recent studies on fault pattern predictability using labeled Petri nets [54] suggest that fault behaviors can be anticipated before their occurrence in discrete event systems. Adapting similar predictive frameworks to IMU-based systems could help identify early signs of adversarial manipulation and enable preemptive countermeasures. Investigating fault prediction models for IMU sensors could be a promising direction for future research, further enhancing the security of cyber–physical systems against adversarial threats.

**Applicability to Other Sensor Types.** The proposed method utilizes mathematical relations between sensor data to provide resilience against signal injection attacks. However, applying this approach to other types of sensors requires several considerations. Different sensors operate based on distinct physical principles, and not all sensor types exhibit strong mathematical dependencies that allow one modality to infer another. Additionally, sensor signal characteristics vary significantly—some sensors measure rapidly changing dynamic data, while others capture relatively stable environmental changes. These differences may require adjustments to our approach to ensure effective estimation and resilience. Nevertheless, exploring inherent relations among different sensor modalities and developing appropriate models could enhance resilience and anomaly detection across various sensing environments. Future research may systematically analyze these relations and investigate adaptation strategies for extending our method to different sensor types.

**Limitations.** While our method provides resilience against temporary disruptions caused by signal injection attacks, it assumes that the attack does not result in permanent hardware damage. Our approach is designed to mitigate transient sensor perturbations rather than irreversible failures.

## 10. Conclusions

IMU sensors, which are used in myriad devices and provide various services, have been demonstrated to be vulnerable to physical signal injection attacks. If such an attack occurs, normal service cannot be provided and even human accidents may occur. Therefore, developing technology that can both detect and respond to signal injection attacks is of utmost importance. In this paper, we propose a first method that detects malicious acts on IMU sensors based on the relation between the gyroscope and magnetometer and also provides long-term resilience. We constructed a mathematical model that estimates gyroscope data from magnetometer data based on the relation between the gyroscope and the magnetometer. This relation has never been considered from a security perspective until now, but we newly applied this relation as a security method to detect and respond to attacks. We also evaluated our proposed method using five open datasets and commercial devices. The results show that our method can detect attacks with a false positive rate of 0.052% and is 99.78% resilient against attacks while consuming only reasonable computational costs. This means that even if a signal injection attack occurred on an IMU sensor, a device would be able to detect the attack and behave in a near-normal state.

## Figures and Tables

**Table 1:** Comparison of existing methods against signal injection attacks.

Method	Prevention	Detection	Resilience	Hardware Required
Secure Filter	**✓**	**✗**	**✗**	**✓**
Shielding	**✓**	**✗**	**✗**	**✓**
Redundant Sensors	**✗**	**✓**	**✗**	**✓**
Dynamic Sampling	**✓**	**✗**	**✗**	**✗**
Sensor Fusion	**✗**	**✓**	**✗**	**✗**
System Model	**✗**	**✓**	**✓** (up to 16s)	**✗**
**Ours**	**✗**	**✓**	**✓** (**at least 1 min**)	**✗**

**Table 2:** Lists of open datasets used in our evaluation.

Dataset	#1	#2	#3	#4	#5
	UMAFall	DSADS	PAMAP2	MHEALTH	REALDISP
Subjects	19	8	9	10	17
Activity	12	19	18	12	33
#(data)	737	45,600	264	240	8820
Loc *	1	5	3	2	9
IMU ^†^	Phone ^§^	MTx	Colibri	Shimmer	MTx
Freq.	200 Hz	25 Hz	100 Hz	50 Hz	50 Hz

* The number of locations where the IMU sensor is attached. ^†^ The IMU sensor contains an accelerometer, a gyroscope, and a magnetometer. ^§^ Samsung S5 and LG G4.

**Table 3:** Gyroscope estimation errors GEE (rad/s).

Dataset	Avg.	Max.	0.25–0.3	0.3–0.35	0.35–
#1	0.0016	0.0137	0%	0%	0%
#2	0.0054	0.0494	0%	0%	0%
#3	0.0395	0.1995	0%	0%	0%
#4	0.0020	0.0104	0%	0%	0%
#5	0.0497	0.3547	0.26%	0.06%	0.01%

**Table 4:** Path correlation values $P_{corr}$.

Dataset	Avg.	Min.	0.7–0.75	0.75–0.8	0.8–0.85	0.85–0.9
#1	0.9999	0.9853	0%	0%	0%	0%
#2	0.9990	0.8115	0%	0%	0.01%	0.03%
#3	0.9813	0.7258	0.38%	1.14%	1.89%	1.52%
#4	0.9983	0.9164	0%	0%	0%	0%
#5	0.9947	0.7156	0.10%	0.17%	0.27%	0.65%

## Data Availability

The data presented in this study are available at the following sources: UMAFall Dataset: https://figshare.com/articles/dataset/UMA_ADL_FALL_Dataset_zip/4214283 (accessed on 24 February 2025), DSADS Dataset: https://archive.ics.uci.edu/dataset/256/daily+and+sports+activities (accessed on 24 February 2025), PAMAP2 Dataset: https://archive.ics.uci.edu/dataset/231/pamap2+physical+activity+monitoring (accessed on 24 February 2025), MHEALTH Dataset: https://archive.ics.uci.edu/dataset/319/mhealth+dataset (accessed on 24 February 2025), REALDISP Dataset: https://archive.ics.uci.edu/dataset/305/realdisp+activity+recognition+dataset (accessed on 24 February 2025).
